# CONTENT AND QUALITY OF CHATGPT AS A SOURCE OF DEMENTIA INFORMATION: COMPARATIVE ANALYSES

**DOI:** 10.1093/geroni/igae098.2990

**Published:** 2024-12-31

**Authors:** Jill Dosso, Jaya Kailley, Payton Angus, Julie Robillard

**Affiliations:** University of British Columbia, Vancouver, British Columbia, Canada; University of British Columbia, Vancouver, British Columbia, Canada; University of British Columbia, Vancouver, British Columbia, Canada; University of British Columbia, Vancouver, British Columbia, Canada

## Abstract

There is significant demand for easily accessible, reliable online health information from older adults and their families (Soong et al., 2020). Online information about Alzheimer’s Disease and dementia ranges in quality from misleading and predatory to supportive and evidence-based (Robillard & Feng, 2016). ChatGPT was released to the public in November 2022 and is a well-known example of a new class of online tools, Large Language Models, which are generative artificial intelligence systems that can produce fluent, coherent answers to users’ questions based on extensive training data. In this work, we use a validated evaluation tool (Robillard et al., 2018) to evaluate the quality and content of information about dementia from ChatGPT-3.5 at two time points (2023, 2024) and from two versions of the model (free GPT-3.5, paid GPT-4). Prompts were developed using questions extracted from the FAQ pages of national dementia organization websites in Canada, USA, and Mexico. We found that all versions of ChatGPT were unbiased, remind the user to speak to a physician, and took an appropriately balanced tone. GPT-4, unlike GPT-3.5, links to specific, identifiable, dated sources for its claims including scientific research. All forms of ChatGPT had a high Flesch-Kincaid Grade Level, indicating moderate readability. Results indicate that ChatGPT, particularly GPT-4, can be a relatively high-quality source of dementia information for non-expert users. However, it is limited in its ability to link users to local resources. Findings can inform older adults, care partners, and healthcare professionals in their decision-making around use of this new tool.

